# Structural and Magnetic Properties of Dimeric Capsule Assemblies Formed by Cyclic Trinuclear Complexes

**DOI:** 10.3390/molecules29184307

**Published:** 2024-09-11

**Authors:** Masahiro Muto, Kousuke Morinaga, Momoko Nishihashi, Yasunori Yamada, Masayuki Koikawa

**Affiliations:** Department of Chemistry and Applied Chemistry, Faculty of Science and Engineering, Saga University, Honjo 1, Saga 840-8502, Japanyyamada@cc.saga-u.ac.jp (Y.Y.)

**Keywords:** dimeric capsule, trinuclear complex, iron(III), manganese(III), ditopic ligand, X-ray structures, magnetic susceptibility

## Abstract

Cyclic trinuclear homo-metal complexes, [{Fe(L^3+2^Br)py}_3_] (**1**) and [{Mn(L^3+2^Br)}_3_(py)_2_ MeOH] (**2**), along with a hetero-metal complex, [FeMn_2_(L^3+2^H)_3_(DMF)_3_] (**3**), were synthesized using asymmetric ditopic ligands (H_3_L^3+2^H: 2-(2-hydroxyphenyl)-6-ol-5-(salicylideneamino)benzoxazole, H_3_L^3+2^Br: 2-(2-hydrox-5-bromoyphenyl)-6-ol-5-(5-bromosalicylideneamino)benzoxazole). The molecular structure of **1** is characterized by a tripod structure with three-fold symmetry, where an enantiomer pair forms a dimeric capsule with dimensions of approximately 3 × 1.6 × 1.6 nm^3^. Complexes **2** and **3**, which lack three-fold symmetry, exhibit similar molecular structures to previously reported complexes with these ligands, but do not form a capsule structure. Magnetic measurements of **1**–**3** reveal the presence of significantly weak antiferromagnetic interactions between the metal ions.

## 1. Introduction

Nanoscale polynuclear transition metal complexes featuring macrocyclic and cage structures have attracted significant interest in materials science and supramolecular chemistry due to their unique molecular structures and properties [1,2,3,4]. These complexes are also of great interest from the perspective of host–guest chemistry, as they enable selective molecular inclusion within their internal spaces of various sizes and properties. Recently, there has been an increase in reports of complexes whose magnetic and electrochemical properties change depending on the guest molecules. This trend is promising for the development of molecular devices that integrate host–guest chemistry with molecular properties [5,6,7,8]. Despite the many polynuclear complexes reported with such molecular inclusion capabilities, few compounds exhibit dimeric capsule structures, highlighting the need for the development of such complexes for nanotechnological applications [9,10].

In our previous study, we reported on cyclic trinuclear Mn(III) and V(V)=O complexes with the general formula [{M(L^3+2^H)(solvent)}_3_], formed using the ditopic ligand H_3_L^3+2^H: 2-(2-hydroxyphenyl)-6-ol-5-(salicylideneamino)benzoxazole, and its homologs [11,12]. These ligands are obtained by reacting the symmetric ligands H_4_L^3+3^H: 4,6-bis[(2-hydroxybenzylidene)imino]benzene-1,3-diol and homologs (H_4_L^3+3^Me and H_4_L^3+3^Br), which contain two tridentate (κ^3^*N*,*O*_2_, κ^3^*N*′,*O*′_2_) coordination sites, with transition metal ions. They are characterized by the asymmetric structures, featuring both a tridentate and a bidentate (κ^3^*N*,*O*_2_, κ^2^*N*′,*O*′) coordination site. The charge balance and composition of the resulting metal complexes suggest that these ligands readily complex with redox-active metal ions in the +3 oxidation state.

**Scheme 1:**
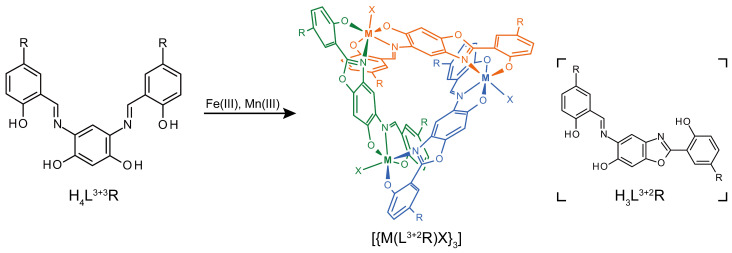
Reaction scheme and chemical diagrams of H_4_L^3+3^R, H_3_L^3+2^R, and [{M(H_3_L^3+2^R)X}_3_] (R = H or Br; M = Fe or Mn; X = pyridine, dimethylformamide, or methanol). The structure of the trinuclear complex is color-coded by mononuclear unit.

In this study, we synthesized new cyclic trinuclear Fe(III)_3_, Mn(III)_3_, and Fe(III)Mn(III)_2_ complexes using these ligands (Scheme 1). Notably, the trinuclear Fe(III)_3_ complex was found to form a dimeric capsule structure, a feature not previously observed in similar complexes. Herein, we present the synthesis, X-ray structural characterization, and magnetic properties of these newly obtained complexes.

## 2. Results and Discussion

The reactions of H_4_L^3+3^Br·HCl with iron(III) chloride or manganese(III) acetate in pyridine yielded the trinuclear iron(III) complex **1** and manganese(III) complex **2**. Elemental analysis data indicated that these complexes have the general composition M(L^3+2^Br)(solvent)*n* (**1**: M = Fe; **2**: M = Mn), with the ligands oxidized to (L^3+2^Br)^3−^. Complex **3** was synthesized with the aim of incorporating Fe ions into an inner space of [Mn_3_(L^3+2^H)_3_(solvent)_3_]. Elemental analysis suggested a composition of M_3_(L^3+2^H)_3_(DMF)_4_(H_2_O)_2_ (M = Fe or Mn). Quantitative analysis of Fe and Mn using inductively coupled plasma atomic emission spectroscopy (ICP-AES) gave a ratio of Fe:Mn = 1:2. This suggests that **3** is not the intended Fe(III)-inclusive trinuclear Mn(III)_3_ complex [Mn(III)_3_]∙∙∙Fe(III)∙∙∙[Mn(III)_3_]. The *ν*(C=N) stretching vibrations observed at 1632–1634 cm^−1^ in the free H_3_L^3+3^R ligands shifted to 1603–1614 cm^−1^ in all complexes, suggesting that each coordination site of the ligands is coordinated to the metal ions as a chelate [13,14]. The phenolic *ν*(C–O) bands were observed at 1215–1310 cm^−1^, similar to those found in [Mn_3_(L^3+2^H)_3_(MeOH)_3_] [11]. Other characteristic bands include δ(C–H) bending vibrations from the pyridine ring at 698 cm^−1^ in **1** and **2**, and *ν*(C=O) vibrations of DMF at 1659 cm^−1^ in **3**.

### 2.1. Crystal Structures

The molecular structures of **1**–**3** were confirmed by single-crystal X-ray structure analyses. The crystallographic data and collection details are summarized in Table 1.

#### 2.1.1. Structure of Complex **1**

**Figure 1:**
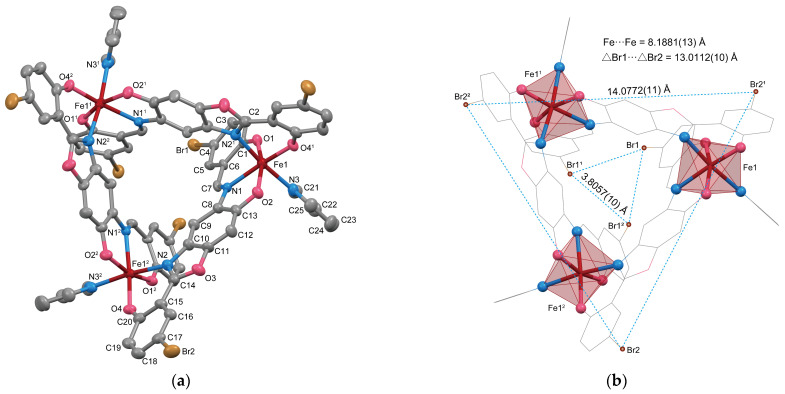
(**a**) Molecular structure of **1** with thermal ellipsoids drawn at a 50% probability level. Symmetry code: (^1^) −*x* + *y*, −*x*, +*z*; (^2^) −*y*, *x* − *y*, +*z*. Hydrogen atoms have been omitted for clarity. (**b**) Coordination polyhedron of Fe with the ligand framework shown as a line drawing.

The molecular structures of **1** are illustrated in Figure 1a, and selected bond lengths and angles are listed in Table 2. Complex **1** is a trinuclear Fe(III) complex featuring a tripodal pyramidal structure with *C*_3_ symmetry. The asymmetric unit consists of [Fe(L^3+2^Br)(py)]∙py. It was confirmed that the ligand H_4_L^3+3^Br is oxidized to H_3_L^3+2^Br, similarly to the previously reported complexes [{Mn(L^3+2^H)(MeOH)}_3_] [11] and [{VO(L^3+2^H)}_3_] [12], which were obtained from the reaction of H_3_L^3+3^H with corresponding metal ions. Each Fe(III) ion is coordinated by the ONO atoms of the tridentate Schiff-base coordination site and the ON atoms of the bidentate benzoxazoline site. The remaining sixth coordination site of the Fe(III) ion is occupied by a monodentate pyridine molecule, resulting in a six-coordinate N_3_O_3_ environment (Figure 1b). The pyridine molecules are coordinated perpendicularly to each face of the tripodal pyramidal structure, and together with the *C*_3_ symmetry, the overall appearance resembles a “pinwheel”. The Fe–O bond lengths are 1.934(4), 1.955(4), and 1.914(3) Å, while the Fe–N bond lengths are 2.166(4), 2.172(4), and 2.198(5) Å. The differences in bond length between Fe–O and Fe–N lead to the distorted Fe(III) geometry. This distortion also affects the diagonal angles, where the O1–Fe1–O2 angle (162.70(15)°) is significantly smaller than the other diagonal angles: 172.92(16)° for N1–Fe1–O4^1^, and 173.32(17)° for N3–Fe1–N2^1^ (Symmetry code ^1^: −*x* + *y*, −*x*, +*z*). This is primary due to the longer Fe1–N1 distance of 2.172(4) Å in the *ONO*-site. The two coordination sites of the ditopic ligand are separated by a benzoxazoline structure. Despite being a trinuclear complex, the distance between the three Fe(III) ions is relatively long, measuring 8.1881(13) Å (Figure 1b), similar to the distances observed in previously reported trinuclear Mn(III) and V(V) complexes [11,12]. The molecular size of the tripodal pyramidal structure can be estimated by examining the positions of the bromine atoms within the ligands, specifically those located at the top (Br1) and bottom (Br2) of the structure. The distances between the bromine atoms are Br1···Br1^1^ = 3.2954(9) Å and Br2···Br2^1^ = 14.0778(14) Å. Therefore, the size of one side of the triangular pyramid structure is estimated to be approximately ~15 Å.

**Figure 2:**
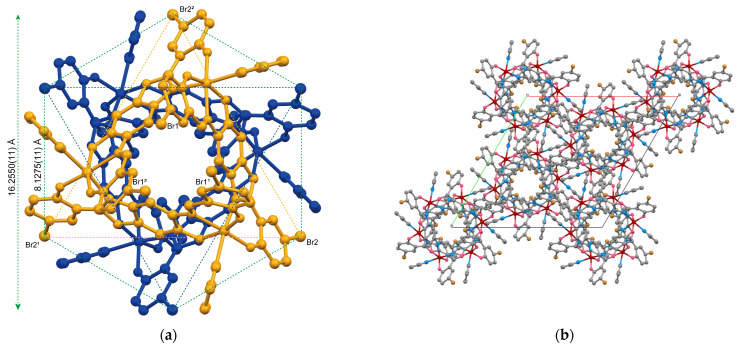
(**a**) Ball-and-stick model for the enantiomeric pair of **1** viewed along the *c* axis. Each enantiomer is color coded. Symmetry code: (^1^) −*x* + *y*, −*x*, +*z*; (^2^) −*y*, *x* − *y*, +*z*. (**b**) Crystal packing view along the *c* axis.

In the previously reported Mn(III) and V(V) complexes with (L^3+2^H)^3−^, enantiomeric pairs loosely aggregate into dimers through π-π stacking. In these structures, the legs of the tripods fit inside each other, filling the internal space of the pyramid. In contrast, in complex **1** (Figure 2a), the enantiomeric pair is symmetrically assembled with the bases of the tripodal pyramids facing each other, leaving the internal space unobstructed and forming a symmetrical dimeric capsule structure. When the position of Br2 in the projection diagram (Figure 2a) is considered as forming a regular hexagon, the outer diameter of the capsule structure is calculated to be 16.2550(10) Å, with each side of the hexagon measuring approximately 8.1275(11) Å. Figure 2b shows the crystal packing viewed along the *c* axis, revealing that the dimeric capsules are aligned in the same direction.

**Figure 3:**
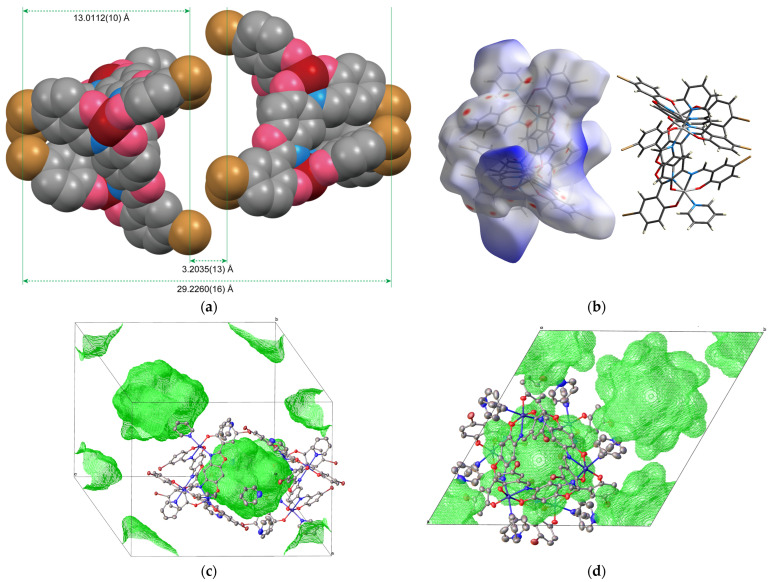
(**a**) Space-filling model of the enantiomeric pair viewed from the *a* axis. Hydrogen atoms and coordinating pyridine molecules have been omitted for clarity. (**b**) Hirshfeld surface of the enantiomeric pair. (**c**) Solvent mask mapping view (green surface) of the unit cell. (**d**) Solvent mask mapping view from the *c* axis.

Figure 3a shows a space-filling model of the dimeric capsule viewed from the *a* axis. In this structure, the triangular plane formed by the three Br1 atoms in each complex is referred to as Plane 1, and the plane formed by the Br2 atoms as Plane 2. The distance between Plane 1 in the enantiomeric pair corresponds to the length of the capsule, while the distance between Plane 2 corresponds to the gap between the hemicapsules. These interplanar distances are 29.2263(16) Å and 3.2043(13) Å, respectively. Considering the van der Waals radii, this molecular assembly is considered to be a nanosized capsule with a length of approximately 3 nm, where the hemicapsules are loosely aggregated. Unfortunately, the quality of the obtained crystals was insufficient to determine details such as the solvent molecules contained within the capsule. Therefore, the interior of the hemicapsule is mapped in blue on the Hirshfeld surface, as shown in Figure 3b [15]. However, since some electron density peaks appear in this space, the structure was refined using the solvent mask method. Figure 3c,d depict the solvent mask surface mapping [16]. The solvent mask is located as a single volume (692 Å^3^) within the inner space of the capsule unit. The estimated electron density within this volume is consistent with the presence of four pyridine molecules in the capsule, suggesting potential applications in host–guest chemistry. The nearest intermolecular Fe1∙∙∙Fe1^3^ distance is 7.7816(14) Å (symmetry code: (^3^) ⅓ − *x*, −⅓ − *y*, ⅔ − *z*), which is slightly shorter than the intramolecular Fe∙∙∙Fe distance of 8.1881(13) Å.

#### 2.1.2. Structure of Complex **2**

**Figure 4:**
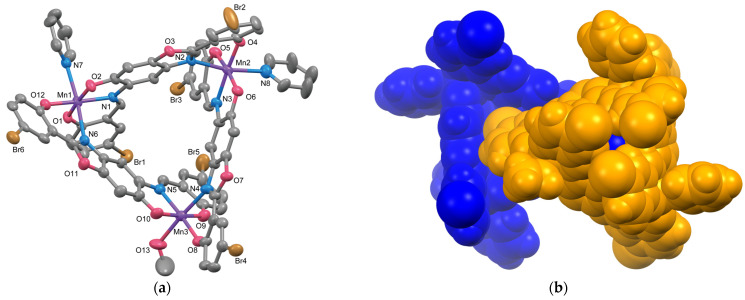
(**a**) Molecular structure of **2** showing thermal ellipsoids drawn at a 50% probability level. Only the disordered pyridine unit with larger occupancy is shown. Hydrogen atoms are omitted for clarity. (**b**) Space-filling model for the enantiomeric pair of **2**. Each monomer unit is color-coded.

The molecular structure of **2** is illustrated in Figure 4a, and the selected bond lengths and angles are listed in Table 3. The pyridine molecule coordinated to Mn2 exhibits rotation disorder, but only the unit with the higher occupancy is shown in Figure 4a. Complex **2** is a trinuclear Mn(III) complex with the same ligand as complex **1**. While its overall appearance is similar to that of **1**, two of the three Mn ions are coordinated by pyridine molecules, and the remaining one is coordinated by methanol, resulting in the absence of the three-fold symmetry, unlike the structure of **1**. The coordination geometries around the Mn atoms resemble those in [{Mn(L^3+2^H)(MeOH)}_3_] [11], featuring an elongated octahedral distortion with the solvent coordination direction as the Jahn–Teller axis. The Jahn–Teller axes are defined as N6–Mn1–N7 [Mn1–N6 2.302(4), Mn1–N7 2.300(4) Å], N2–Mn2–N8 [Mn2–N2 2.254(4), Mn2–N8 2.296(5) Å], and N4–Mn3–O13 [Mn3–N4 2.255(4), Mn3–O13 2.229(4) Å]. Mn3, which is coordinated by methanol, has a slightly shorter Jahn–Teller axis compared to the other Mn atoms. The bond distances between Mn and the coordinating atoms in the equatorial planes are nearly identical, ranging from 1.883(4) to 1.905(3) Å. Figure 4b shows the overlap of the enantiomer pair. Unlike complex **1**, no capsule structure is formed, and the phenol moiety (a phenyl ring with Br4 as a substituent) bonded to Mn3 fits into the cavity of the paired unit, similar to previously reported trinuclear complexes with (L^3+2^H)^3−^.

#### 2.1.3. Structure of Complex **3**

**Figure 5:**
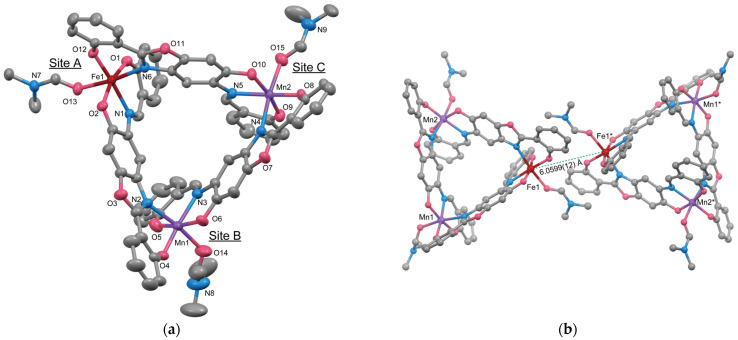
(**a**) Molecular structure of **3** showing thermal ellipsoids drawn at a 30% probability level. Hydrogen atoms have been omitted for clarity. (**b**) Ball-and-stick model of the nearest trinuclear units. Symmetry code: (*) 1 − *x*, −*y*, 1 − *z*.

The molecular structure of **3** is illustrated in Figure 5a. Complex **3** is a trinuclear complex with a tripodal pyramid structure similar to that of **1** and **2**. ICP analysis clearly indicates that Complex **3** contains Fe and Mn in a 1:2 ratio, suggesting that one of the three metal atoms is Fe and the other two are Mn. Table 4 shows the convergence results of *R* values and equivalent isotropic displacement parameters (*U*_eq_) for each metal atom when the Fe atom is assigned to each site for three metal positions (Site A, B, and C) and refined. The *R* value and *U*_eq_ of Fe is smallest when Fe is located at Site A. Additionally, the relative balance of *U*_eq_ values for the three sites shows minimal deviation when Fe is positioned at Site A. Considering all these factors, the structure shown in Figure 5a, where Fe is placed at Site A, is considered the most appropriate. The environment of Site A in **3** is distinct, particularly in terms of its proximity to the neighboring molecule. Figure 5b shows two molecules with the closest Site A. The intermolecular distance between Site A positions is 6.0599(12) Å, which is very close to the neighboring molecule and considerably shorter than the closest intermolecular metal-to-metal distances observed in complexes **1** (7.7816(14) Å) and **2** (7.5829(19) Å). The aspect of crystal packing also suggests that the environment of site A in **3** is significantly different from the other two metal sites.

The selected bond lengths and angles are listed in Table 5. Considering the direction of the coordinated solvent molecules as the axial axis, the axial distances are as follows: for Fe1, Fe1–O13 = 2.1065(19) and Fe1–N6 = 2.1958(19); for Mn1, Mn1–O14 = 2.208(4) and Mn1–N2 = 2.215(2); and for Mn2, Mn2–O15 = 2.168(2) and Mn2–N4 = 2.222(2) Å. Since the axial bonds length of Mn1 and Mn2 are approximately 0.1 Å longer than those of Fe1, indicating Jahn–Teller distortion, the assignment of Fe and Mn appear to be appropriate. The intramolecular metal-to-metal distances range from 8.0924(14) to 8.1642(14) Å, which is consistent with those observed in the analogous trinuclear complexes [11,12].

### 2.2. Magnetic Properties

**Figure 6:**
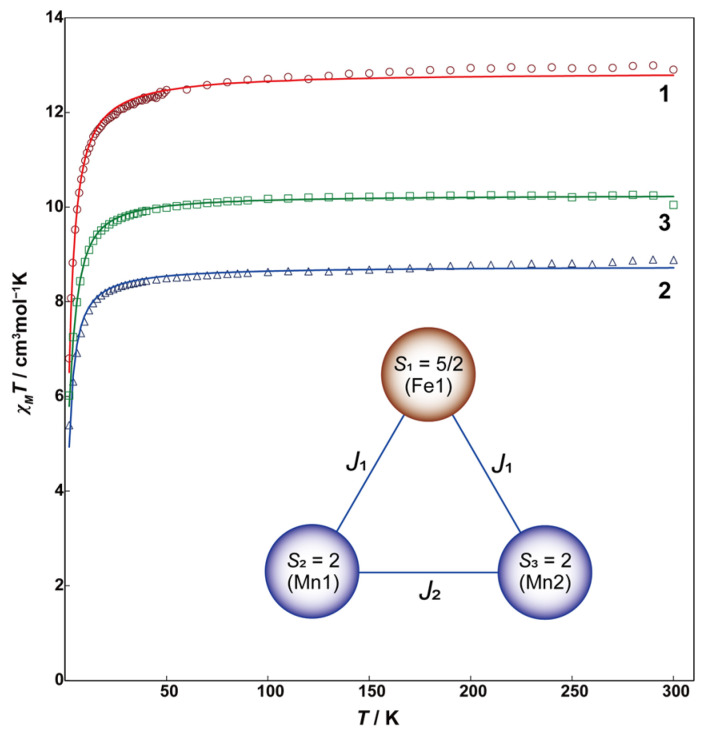
Temperature dependence of *χ*_M_*T* vs. *T* plots of **1** (red circles), **2** (blue triangles), and **3** (green squares). Solid lines are drawn with the best-fitted parameter values described in the text. The inset figure is the magnetic coupling scheme for **3**.

Magnetic susceptibility measurements for **1**–**3** were performed using a SQUID (superconducting quantum interference device) magnetometer. The temperature dependence of magnetic susceptibilities was measured in the temperature range of 2 to 300 K. Figure 6 shows *χ*_M_*T* vs. *T* plots for **1** (bred), **2** (blue), and **3** (green). The *χ*_M_*T* values for **1**–**3** at 300 K are 12.91, 8.89, and 10.05 cm^3^mol^−1^K, respectively, corresponding to three magnetically independent high-spin Fe(III) ions for **1**, three Mn(III) ions for **2**, and one Fe(III) ion and two Mn(III) ions for **3**. The *χ*_M_*T* values remain almost constant from 300 K to 50 K, then decrease with decreasing temperature, reaching 6.80, 5.39, and 6.02 cm^3^mol^−1^K at 2 K, respectively. These behaviors indicate the presence of fairly weak antiferromagnetic interactions between three metal ions. The behaviors of **1** and **2** were simulated using a triangular spin model for *S* = 5/2 for **1** and *S* = 2 for **2**. The spin Hamiltonian is given by Equation (1).

*H* = −2*J*(***S***_1_***S***_2_ + ***S***_2_***S***_3_ + ***S***_3_***S***_1_) (1)

The calculation was performed by the PHI program [17]. The obtained best-fitting parameters for **1** and **2** are *J* = −0.089 cm^−1^, *g* = 1.98 for **1**, and *J* = −0.105 cm^−1^, *g* = 1.97 for **2**. These *J* values indicate that the antiferromagnetic interactions in **1** and **2** are notably weak. The intrametallic distances are quite long, exceeding 8 Å in both complexes, but there appears to be a slight interaction mediated by the π-conjugated system of the ditopic ligand (L^3+2^Br)^3−^.

For Complex **3**, the analysis was performed using the spin Hamiltonian for a heterometallic trinuclear complex, as represented by Equation (2) and the coupling scheme shown in the inset of Figure 5 [18].

*H* = −2*J*_1_(***S***_1_***S***_2_ + ***S***_1_***S***_3_) − 2*J*_2_***S***_2_***S***_3_ (2)

The parameters obtained for **3** are as follows: *J*_1_ = −0.213 cm^−1^, *J*_2_ = −0.390 cm^−1^, *g*_Fe(III)_ = 2.00, *g*_Mn(III)_ = 1.98. Although ferromagnetism can be observed in heterometallic complexes, the magnetic interaction tendency of **3** is nearly the same as that of the homometallic complexes **1** and **2**. The antiferromagnetic interactions between the metal ions in the present complexes **1**–**3** are relatively weak, which allows these complexes to maintain magnetism over a wide temperature range. Therefore, they have potential applications as magnetically responsive capsule materials.

## 3. Materials and Methods

### 3.1. Materials

All reagents and solvents were purchased and used without further purification unless otherwise specified. Methanol was purified by distillation over magnesium turnings. The symmetric ligands, H_4_L^3+3^H·HCl and H_4_L^3+3^Br·HCl, were prepared according to the methods reported in the literature [11].

### 3.2. Preparation

#### 3.2.1. [{Fe(L^3+2^Br)py}_3_] (**1**)

A methanol solution (10 mL) of iron(III) nitrate enneahydrate (0.040 g, 0.1 mmol) was added to a pyridine solution (5 mL) of H_4_L^3+3^Br·HCl (0.058 g, 0.1 mmol) with stirring. The resulting dark brown solution was filtered, and the filtrate was allowed to stand for two weeks at room temperature. Single crystals suitable for X-ray crystallography were obtained from the filtrate. **1**·4py: Yield 0.028 g (39%). C_95_H_62_Br_6_Fe_3_N_13_O_12_ (2224.55): calcd. C 51.3, H 2.8, N 8.2%; found C 50.9, H 2.8, N 8.1%. IR (ATR) [cm^−1^]: 1603(s), 1518(m), 1460–1447(vs), 1310(vs), 1246(s), 1215(m), 1173(s), 1144(s), 827(s), 694(vs), 646(s), 559(s), 515(m), 461(s).

#### 3.2.2. [Mn_3_(L^3+2^Br)_3_(py)_2_CH_3_OH] (**2**)

A methanol solution (20 mL) of manganese(III) acetate dihydrate (0.053 g, 0.2 mmol) was added to a pyridine solution (10 mL) of H_4_L^3+3^Br·HCl (0.108 g, 0.1 mmol) with stirring. The resulting dark brown solution was filtered, and the filtrate was allowed to stand for 7 h at room temperature. Single crystals suitable for X-ray crystallography were obtained. **2**·2H_2_O: Yield 0.045 g (36%). C_71_H_45_Br_6_Mn_3_N_8_O_15_ (1894.4): calcd. C 45.0, H 2.4, N 5.9; found C 45.3, H 2.2, N 5.7%. IR (ATR) [cm^−1^]: 1608(m), 1510(m), 1450–1443(vs), 1352(m), 1310(s), 1292(s), 1236(s), 1215(m), 1171(s), 1138(s), 825(s), 698(vs), 644(s), 567(s), 521(m), 465(m).

#### 3.2.3. [FeMn_2_(L^3+2^H)_3_(DMF)_3_] (**3**)

Triethylamine (0.031 g, 0.3 mmol) was added dropwise to a mixture of manganese(III) acetate dihydrate (0.080 g, 0.3 mmol) and H_4_L^3+3^H·HCl (0.115 g, 0.3 mmol) in 5 mL of DMF with stirring. After 2 h, a 5 mL DMF solution of iron(III) chloride hexahydrate (0.014 g, 0.05 mmol) was added to the resulting dark brown solution, followed by filtration. Single crystals suitable for X-ray crystallography were obtained by the diffusion of diethyl ether into the filtrate. **3**·DMF·2H_2_O: Yield 0.046 g (60%). C_72_H_65_FeMn_2_N_10_O_18_ (1524.06): calcd. C 56.7, H 4.3, N 9.2, Fe 3.7, Mn 7.2%; found C 56.8, H 4.0, N 9.2, Fe 3.8, Mn 7.2%. IR (ATR) [cm^−1^]: 1659(s), 1614(s), 1599(vs), 1520(s), 1462(s), 1448(vs), 1377(s), 1339(s), 1323(s), 1247(m), 1142(s), 980(m), 825(s), 748(s), 652(m), 578(m), 517(m), 460(w).

### 3.3. Physical Measurements

Elemental analyses for C, H and N were performed at the Elemental Analysis Service Center, Kyushu University. Analyses of iron and manganese were made on a Sequential Plasma Spectrometer ICPS-8100 (Shimadzu Co., Ltd., Kyoto, Japan). Infrared spectra were recorded using a VERTEX70-S FT-IR Spectrometer (Bruker, Corp., Billerica, MA, USA) with the attenuated total reflection (ATR) method. The IR spectra for **1**–**3** are presented in Figures S1–S3. Magnetic susceptibilities were measured using a Quantum Design MPMS-XL5R SQUID susceptometer (Quantum Design, Inc., San Diego, CA, USA) under an applied magnetic field of 0.1 T, in the temperature range 2–300 K. The susceptibilities were corrected for the diamagnetism of the constituent atoms using Pascal’s constant [19].

### 3.4. X-ray Crystallography

Diffraction data were collected using a Vari-Max Saturn CCD 724 diffractometer (Rigaku, Corp., Tokyo, Japan) using graphite monochromated Mo Ka radiation (λ = 0.71069 Å) at the Analytical Research Center for Experimental Sciences, Saga University. Data were acquired using CrystalClear [20]. Data processing for **1** was performed with CrysAlisPro [21], while CrystalClear was used for **2** and **3**. A multi-scan correction for absorption was applied. The structures were solved by direct methods (ShelXT) and expanded using Fourier techniques [22]. Olex2 1.5 was used as an interface to the ShelX program package [23]. Non-hydrogen atoms were refined anisotropically, while hydrogen atoms were placed geometrically in calculated positions and refined with a riding model. Solvent masks were calculated to account for disordered solvent molecules in all complexes [24]. The numbers of electrons in the estimated solvent mask volumes within one void per unit cell are as follows: 534 electrons in a volume of 2079 Å^3^ for **1**, 223 electrons in a volume of 1034 Å^3^ for **2**, and 108 electrons in a volume of 1282 Å^3^ for **3**. These values correspond to the presence of 2 pyridine, 2.5 pyridine, and 1.5 DMF molecules per formula unit, respectively. The final cycle of full-matrix least-squares refinement on *F*_2_ using ShelXL [25] was based on observed reflections and variable parameters, converging with unweighted and weighted agreement factors of *R* and *R_w_*. Molecular structure drawings were performed using Mercury 2022 [26]. The crystallographic data and collection details are summarized in Table 1.

## 4. Conclusions

The present study has demonstrated that the asymmetric ditopic ligands H_3_L^3+2^Br and H_3_L^3+2^H promote the formation of cyclic trinuclear Fe(III) and Mn(III) complexes **1** and **2**, as well as the heteronuclear Fe(III)Mn(III)_2_ complex **3**. In these complexes, the metal atoms adopt six-coordinate geometries, and the molecular structures are characterized as tripodal pyramids with small cavities. Among these complexes, Complex **1**, which is the only one with three-fold symmetry, forms a dimeric capsule structure with approximate dimensions of 3 × 1.6 × 1.6 nm^3^, where the enantiomeric pairs overlap with the symmetry center aligned. Due to the estimated internal space capable of encapsulating four pyridine molecules, Complex **1** could potentially be utilized in host–guest chemistry. Furthermore, since these trinuclear complexes are paramagnetic over a wide temperature range, they are expected to have applications in systems such as magnetic drug delivery systems (MDDS), using them as hemicapsules.

## Figures and Tables

**Table 1:** Crystallographic data and refinement parameters.

	1	2	3
Empirical formula	C_100_H_67_Br_6_Fe_3_N_14_O_12_	C_86_H_56_Br_6_Mn_3_N_11_O_13_	C_76.5_H_71.5_FeMn_2_N_11.5_O_17.5_
Formula weight	2303.68	2095.69	1597.68
Temperature/K	113.15	113.15	293.15
Crystal system	trigonal	triclinic	triclinic
Space group	$R\bar{3}$	$P\bar{1}$	$P\bar{1}$
*a*/Å	22.3202(8)	15.995(3)	14.417(3)
*b*/Å	22.3202(8)	16.278(3)	15.001(3)
*c*/Å	32.1552(10)	17.829(4)	20.820(5)
*α*/°	90	78.264(9)	102.622(3)
*β*/°	90	83.109(9)	97.115(2)
*γ*/°	120	70.544(7)	94.088(3)
*V*/Å^3^	13,873.2(11)	4278.8(15)	2495.7(8)
*Z*	6	2	2
*D*_calc_/g cm^−3^	1.654	1.627	1.223
m(MoKa)/mm^−1^	3.128	3.306	0.519
*F*(0 0 0)	6894.0	2076.0	1654.0
Crystal dimensions/mm^3^	0.1 × 0.074 × 0.062	0.21 × 0.17 × 0.12	0.47 × 0.39 × 0.34
Radiation	MoKα (λ = 0.71073)	MoKα (λ = 0.71075)	MoKα (λ = 0.71075)
2*q* range for data collection/°	3.3 to 61.906	6.014 to 54.954	6.078 to 54.918
Reflections collected	43,139	35,546	35,454
Independent reflections	9022	18,867	19,007
Data/Restraints/Params	9022/0/370	18,867/126/1014	19,007/30/918
Goodness of fit indicator	0.990	0.983	1.032
*R* indices [*I* > 2.00s(*I*)]	*R*_1_ = 0.0785	*R*_1_ = 0.0602	*R*_1_ = 0.0624
	*wR*_2_ = 0.1278	*wR*_2_ = 0.1461	*wR*_2_ = 0.1842
*R* indices (all data)	*R*_1_ = 0.1852	*R*_1_ = 0.0982	*R*_1_ = 0.0704
	*wR*_2_ = 0.1593	*wR*_2_ = 0.1647	*wR*_2_ = 0.1947
Largest diff. peak, hole/e Å^−3^	1.03, −0.76	1.74, −0.90	0.43, −0.65
CCDC deposition number	2379560	2379559	2379558

**Table 2:** Selected bond distances and angles of **1**.

Bond	Distance/Å	Angle	Angle/°
Fe1–O1	1.934(4)	O1–Fe1–O2	162.70(15)
Fe1–O2	1.955(4)	O4^1^–Fe1–N1	172.92(16)
Fe1–O4^1^	1.914(3)	N2^1^–Fe1–N3	173.32(17)
Fe1–N1	2.172(4)	O1–Fe1–N1	84.56(15)
Fe1–N2′	2.166(4)	O2–Fe1–N1	78.14(15)
Fe1–N3	2.198(5)	O4^1^–Fe1–N2^1^	85.49(16)
Fe1···Fe1^1^	8.1881(13)		

Symmetry code: (^1^) −*x* + *y*, −*x*, +*z*.

**Table 3:** Selected bond distances and angles of **2**.

Bond	Distance/Å	Angle	Angle/°
Mn1–O1	1.894(3)	O1–Mn1–O2	173.96(15)
Mn1–O2	1.895(3)	O12–Mn1–N1	175.10(15)
Mn1–O12	1.886(3)	N6–Mn1–N7	173.84(15)
Mn1–N1	2.014(4)	O1–Mn1–N1	90.22(15)
Mn1–N6	2.302(4)	O2–Mn1–N1	83.75(15)
Mn1–N7	2.300(4)	O12–Mn1–N6	83.91(15)
Mn2–O4	1.896(3)	O5–Mn2–O6	172.45(14)
Mn2–O5	1.899(3)	O4–Mn2–N3	173.33(15)
Mn2–O6	1.905(3)	N2–Mn2–N8	169.49(16)
Mn2–N2	2.254(4)	O5–Mn2–N3	89.97(15)
Mn2–N3	2.002(4)	O6–Mn2–N3	82.51(15)
Mn2–N8	2.296(5)	O4–Mn2–N2	83.00(15)
Mn3–O8	1.883(4)	O9–Mn3–O10	174.40(16)
Mn3–O9	1.903(4)	O8–Mn3–N5	174.92(18)
Mn3–O10	1.892(4)	O13–Mn3–N4	175.88(17)
Mn3–O13	2.229(4)	O9–Mn3–N5	91.08(17)
Mn3–N4	2.255(4)	O10–Mn3–N5	83.31(17)
Mn3–N5	2.011(4)	O8–Mn3–N4	84.70(15)
Mn1···Mn2	8.1311(16)	Mn1···Mn2···Mn3	60.478(16)
Mn1···Mn3	8.1870(19)	Mn1···Mn3···Mn2	59.794(12)
Mn2···Mn3	8.1255(17)	Mn2···Mn1···Mn3	59.727(14)

**Table 4:** *R* and *U*_eq_ values after final refinement according to the position of Fe (Site A–C).

Fe Position	*R* Value		*U*_eq_/Å^2^	
		A	B	C
Site A	0.0624	0.053 (Fe)	0.069 (Mn)	0.055 (Mn)
Site B	0.0632	0.050 (Mn)	0.073 (Fe)	0.056 (Mn)
Site C	0.0635	0.049 (Mn)	0.069 (Mn)	0.059 (Fe)

**Table 5:** Selected bond distances and angles of **3**.

Bond	Distance/Å	Angle	Angle/°
Fe1–O1	1.9290(18)	O1–Fe1–O2	166.28(7)
Fe1–O2	1.9438(18)	O12–Fe1–N1	173.56(8)
Fe1–O12	1.9035(17)	O13–Fe1–N6	173.57(8)
Fe1–O13	2.1065(19)	O1–Fe1–N1	87.05(7)
Fe1–N1	2.0927(19)	O2–Fe1–N1	80.09(7)
Fe1–N6	2.1958(19)	O12–Fe1–N6	84.21(7)
Mn1–O4	1.898(2)	O5–Mn1–O6	169.68(9)
Mn1–O5	1.906(2)	O4–Mn1–N3	172.99(10)
Mn1–O6	1.910(2)	O14–Mn1–N2	171.52(13)
Mn1–O14	2.208(4)	O5–Mn1–N3	88.43(10)
Mn1–N2	2.215(2)	O6–Mn1–N3	81.27(9)
Mn1–N3	2.066(2)	O4–Mn1–N2	84.98(9)
Mn2–O8	1.9024(19)	O9–Mn2–O10	168.70(8)
Mn2–O9	1.916(2)	O8–Mn2–N5	173.32(8)
Mn2–O10	1.9322(19)	O15–Mn2–N4	173.58(8)
Mn2–O15	2.168(2)	O9–Mn2–N5	87.88(8)
Mn2–N4	2.222(2)	O10–Mn2–N5	80.85(7)
Mn2–N5	2.074(2)	O8–Mn2–N4	83.90(8)
Fe1···Mn1	8.1506(14)	Fe1···Mn1···Mn2	59.473(14)
Fe1···Mn2	8.0924(14)	Fe1···Mn2···Mn1	60.180(8)
Mn1···Mn2	8.1642(14)	Mn1···Fe1···Mn2	60.347(9)
Fe1···Fe1 *	6.0599(12)		

Symmetry code: (*) 1 − *x*, −*y*, 1 − *z*.

## Data Availability

The data that support the findings of this study are available from the author upon reasonable request. The data are not publicly available due to privacy.

## Supplementary Materials

The following supporting information can be downloaded at: https://www.mdpi.com/article/10.3390/molecules29184307/s1, Figure S1. IR spectrum of **1**. Figure S2. IR spectrum of **2**. Figure S3. IR spectrum of **3**.
