# Supplementary material for: Structural and Magnetic Properties of Dimeric Capsule Assemblies Formed by Cyclic Trinuclear Complexes
Source: Molecules. 2024 Sep 11;29(18):4307. doi: 10.3390/molecules29184307 (PMC11433658; doi:10.3390/molecules29184307)

# Supplementary Materials

# Structural and Magnetic Properties of Dimeric Capsule Assemblies Formed by Cyclic Trinuclear Complexes

**Masahiro Muto, Kousuke Morinaga, Momoko Nishihashi, Yasunori Yamada, and Masayuki Koikawa***

Department of Chemistry and Applied Chemistry, Faculty of Science and Engineering, Saga University, Honjo 1, Saga 840-8502, Japan

* Correspondence: koikawa@cc.saga-u.ac.jp

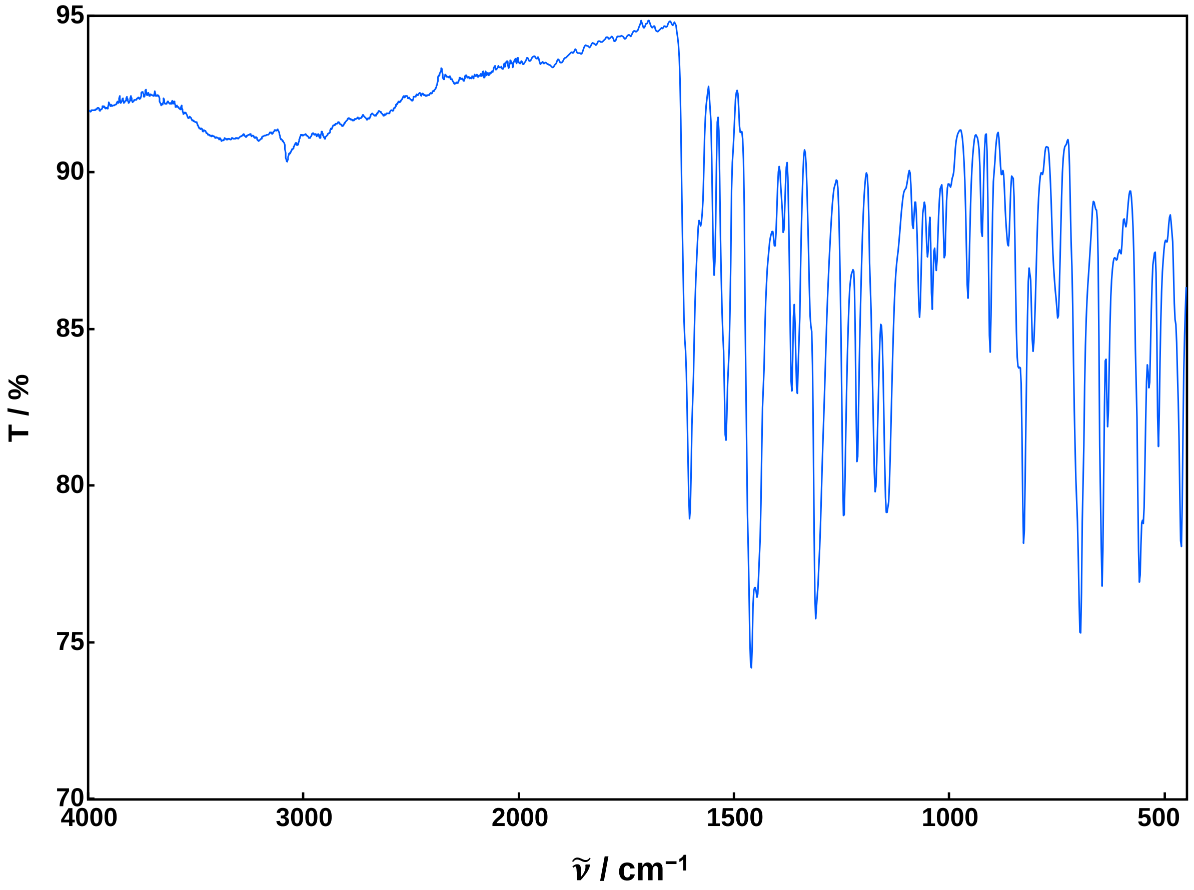


**Figure S1.** IR spectrum of **1**.

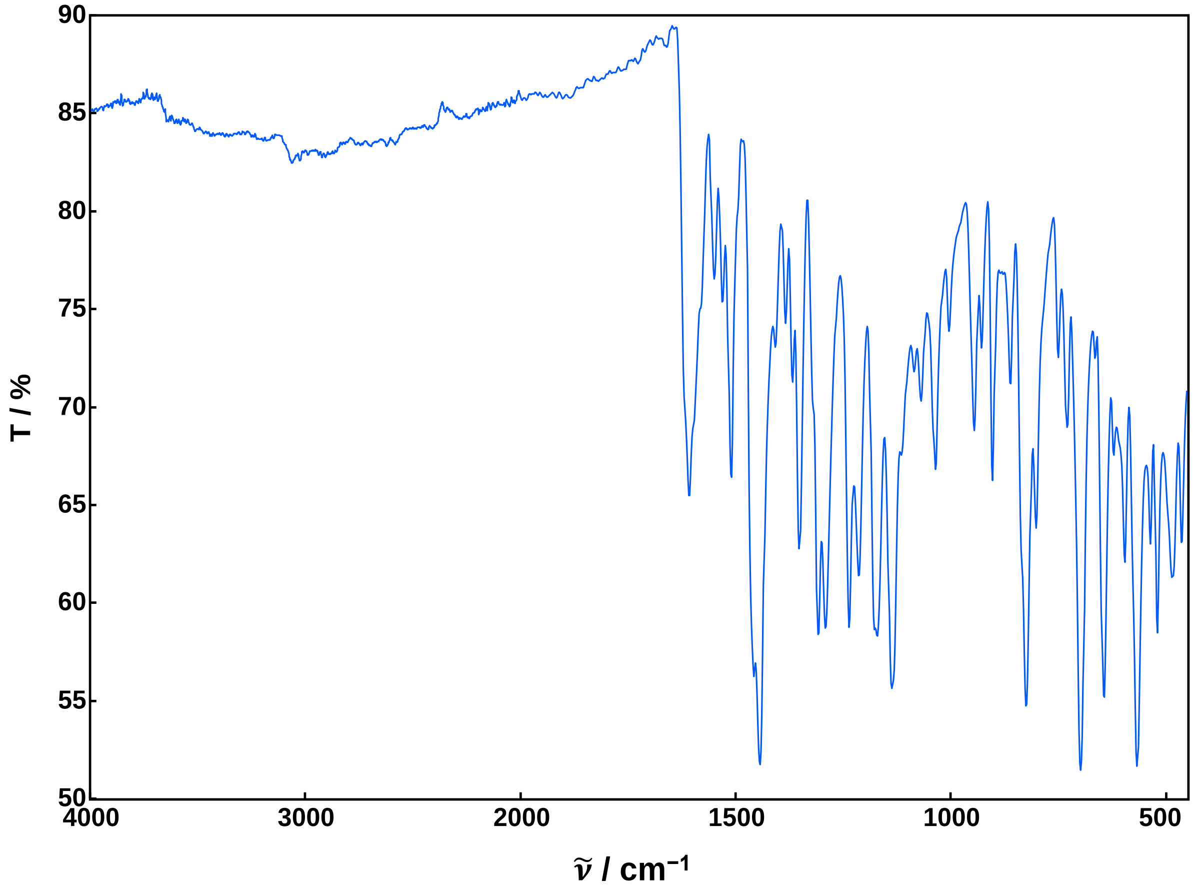


**Figure S2.** IR spectrum of **2**.

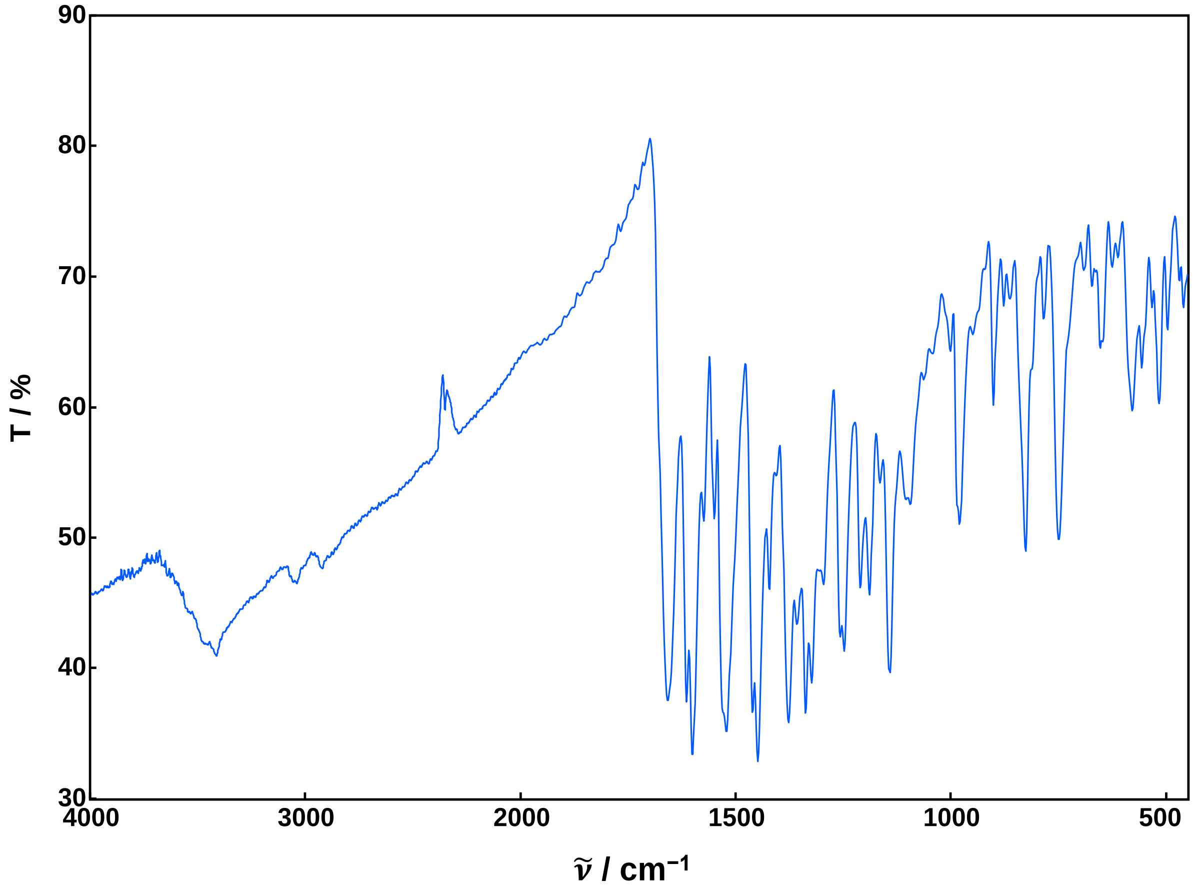


**Figure S3.** IR spectrum of **3**. The upward peak around 2300 cm$^{-1}$ is caused by a background correction error.

# checkCIF/PLATON report

Structure factors have been supplied for datablock(s) fe3lbr3py3

THIS REPORT IS FOR GUIDANCE ONLY. IF USED AS PART OF A REVIEW PROCEDURE FOR PUBLICATION, IT SHOULD NOT REPLACE THE EXPERTISE OF AN EXPERIENCED CRYSTALLOGRAPHIC REFEREE.

No syntax errors found. CIF dictionary Interpreting this report

# Datablock: fe3lbr3py3

---

Bond precision: C-C = 0.0091 A Wavelength=0.71073

| | | | |
|---|---|---|---|
| Cell: | a=22.3202(8) | b=22.3202(8) | c=32.1552(10) |
| | alpha=90 | beta=90 | gamma=120 |
| Temperature: | 113 K | | |

| | Calculated | Reported |
|---|---|---|
| Volume | 13873.2(11) | 13873.2(11) |
| Space group | R -3 | R -3 |
| Hall group | -R 3 | -R 3 |
| Moiety formula | C75 H42 Br6 Fe3 N9 O12, 3(C5 H5 N) [+ solvent] | C75 H42 Br6 Fe3 N9 O12, 3(C5 H5 N), 2[C5H5N] |
| Sum formula | C90 H57 Br6 Fe3 N12 O12 [+ solvent] | C100 H67 Br6 Fe3 N14 O12 |
| Mr | 2145.43 | 2303.68 |
| Dx,g cm-3 | 1.541 | 1.654 |
| Z | 6 | 6 |
| Mu (mm-1) | 3.121 | 3.128 |
| F000 | 6390.0 | 6894.0 |
| F000’ | 6388.34 | |
| h,k,lmax | 32,32,46 | 32,31,45 |
| Nref | 9811 | 9022 |
| Tmin,Tmax | 0.757,0.824 | 0.787,0.847 |
| Tmin’ | 0.731 | |

Correction method= # Reported T Limits: Tmin=0.787 Tmax=0.847
AbsCorr = NUMERICAL

Data completeness= 0.920 Theta(max)= 30.953

R(reflections)= 0.0785( 4134)    wR2(reflections)= 0.1593( 9022)

S = 0.990    Npar= 370

---

The following ALERTS were generated. Each ALERT has the format
**test-name_ALERT_alert-type_alert-level.**
Click on the hyperlinks for more details of the test.

---

## Alert level C

```
ABSTY02_ALERT_1_C  An _exptl_absorpt_correction_type has been given without
            a literature citation. This should be contained in the
            _exptl_absorpt_process_details field.
            Absorption correction given as numerical
RINTA01_ALERT_3_C  The value of Rint is greater than 0.12
            Rint given   0.160
PLAT020_ALERT_3_C The Value of Rint is Greater Than 0.12 .........      0.160 Report
PLAT026_ALERT_3_C Ratio Observed / Unique Reflections (too) Low ..        46% Check
PLAT243_ALERT_4_C High   'Solvent' Ueq as Compared to Neighbors of         N4 Check
PLAT341_ALERT_3_C Low Bond Precision on  C-C Bonds ...............    0.00914 Ang.
PLAT905_ALERT_3_C Negative K value in the Analysis of Variance ...     -1.040 Report
PLAT906_ALERT_3_C Large K Value in the Analysis of Variance ......      2.792 Check
PLAT977_ALERT_2_C Check Negative Difference Density on H29       .      -0.36 eA-3
```

---

## Alert level G

```
FORMU01_ALERT_2_G  There is a discrepancy between the atom counts in the
            _chemical_formula_sum and the formula from the _atom_site* data.
            Atom count from _chemical_formula_sum:C100 H67 Br6 Fe3 N14 O12
            Atom count from the _atom_site data:  C90 H57 Br6 Fe3 N12 O12
CELLZ01_ALERT_1_G Difference between formula and atom_site contents detected.
CELLZ01_ALERT_1_G ALERT: Large difference may be due to a
            symmetry error - see SYMMG tests
           From the CIF: _cell_formula_units_Z    6
           From the CIF: _chemical_formula_sum  C100 H67 Br6 Fe3 N14 O12
           TEST: Compare cell contents of formula and atom_site data

           atom    Z*formula  cif sites diff
           C         600.00    540.00   60.00
           H         402.00    342.00   60.00
           Br         36.00     36.00    0.00
           Fe         18.00     18.00    0.00
           N          84.00     72.00   12.00
           O          72.00     72.00    0.00
PLAT041_ALERT_1_G Calc. and Reported SumFormula    Strings  Differ     Please Check
             Calc: C90 H57 Br6 Fe3 N12 O12
             Rep.: C100 H67 Br6 Fe3 N14 O12
PLAT042_ALERT_1_G Calc. and Reported MoietyFormula Strings  Differ     Please Check
             Calc: C75 H42 Br6 Fe3 N9 O12, 3(C5 H5 N)
             Rep.: C75 H42 Br6 Fe3 N9 O12, 3(C5 H5 N), 2[C5H5N]
PLAT398_ALERT_2_G Deviating  C-O-C   Angle From 120 for O3       .      105.8 Degree
PLAT606_ALERT_4_G Solvent Accessible VOID(S) in Structure ........          ! Info
PLAT794_ALERT_5_G Tentative Bond Valency for Fe1        (III)     .       2.99 Info
PLAT868_ALERT_4_G ALERTS Due to the Use of _smtbx_masks Suppressed          ! Info
PLAT910_ALERT_3_G Missing # of FCF Reflection(s) Below Theta(Min).          1 Note
```

```
             -1  1  1,
PLAT912_ALERT_4_G Missing # of FCF Reflections Above STh/L=  0.600        782 Note
PLAT941_ALERT_3_G Average HKL Measurement Multiplicity ..........         4.8 Low
PLAT969_ALERT_5_G The 'Henn et al.' R-Factor-gap value ..........       1.956 Note
             Predicted wR2: Based on SigI**2  8.14 or SHELX Weight 16.10
PLAT978_ALERT_2_G Number C-C Bonds with Positive Residual Density.          0 Info
```

---

```
   0 ALERT level A = Most likely a serious problem - resolve or explain
   0 ALERT level B = A potentially serious problem, consider carefully
   9 ALERT level C = Check. Ensure it is not caused by an omission or oversight
  14 ALERT level G = General information/check it is not something unexpected

   5 ALERT type 1 CIF construction/syntax error, inconsistent or missing data
   4 ALERT type 2 Indicator that the structure model may be wrong or deficient
   8 ALERT type 3 Indicator that the structure quality may be low
   4 ALERT type 4 Improvement, methodology, query or suggestion
   2 ALERT type 5 Informative message, check
```

---

It is advisable to attempt to resolve as many as possible of the alerts in all categories. Often the minor alerts point to easily fixed oversights, errors and omissions in your CIF or refinement strategy, so attention to these fine details can be worthwhile. In order to resolve some of the more serious problems it may be necessary to carry out additional measurements or structure refinements. However, the purpose of your study may justify the reported deviations and the more serious of these should normally be commented upon in the discussion or experimental section of a paper or in the "special_details" fields of the CIF. checkCIF was carefully designed to identify outliers and unusual parameters, but every test has its limitations and alerts that are not important in a particular case may appear. Conversely, the absence of alerts does not guarantee there are no aspects of the results needing attention. It is up to the individual to critically assess their own results and, if necessary, seek expert advice.

**Publication of your CIF in IUCr journals**

A basic structural check has been run on your CIF. These basic checks will be run on all CIFs submitted for publication in IUCr journals (*Acta Crystallographica*, *Journal of Applied Crystallography*, *Journal of Synchrotron Radiation*); however, if you intend to submit to *Acta Crystallographica Section C* or *E* or *IUCrData*, you should make sure that full publication checks are run on the final version of your CIF prior to submission.

**Publication of your CIF in other journals**

Please refer to the *Notes for Authors* of the relevant journal for any special instructions relating to CIF submission.

---

**PLATON version of 13/05/2024; check.def file version of 04/05/2024**

**Datablock fe3lbr3py3** - ellipsoid plot

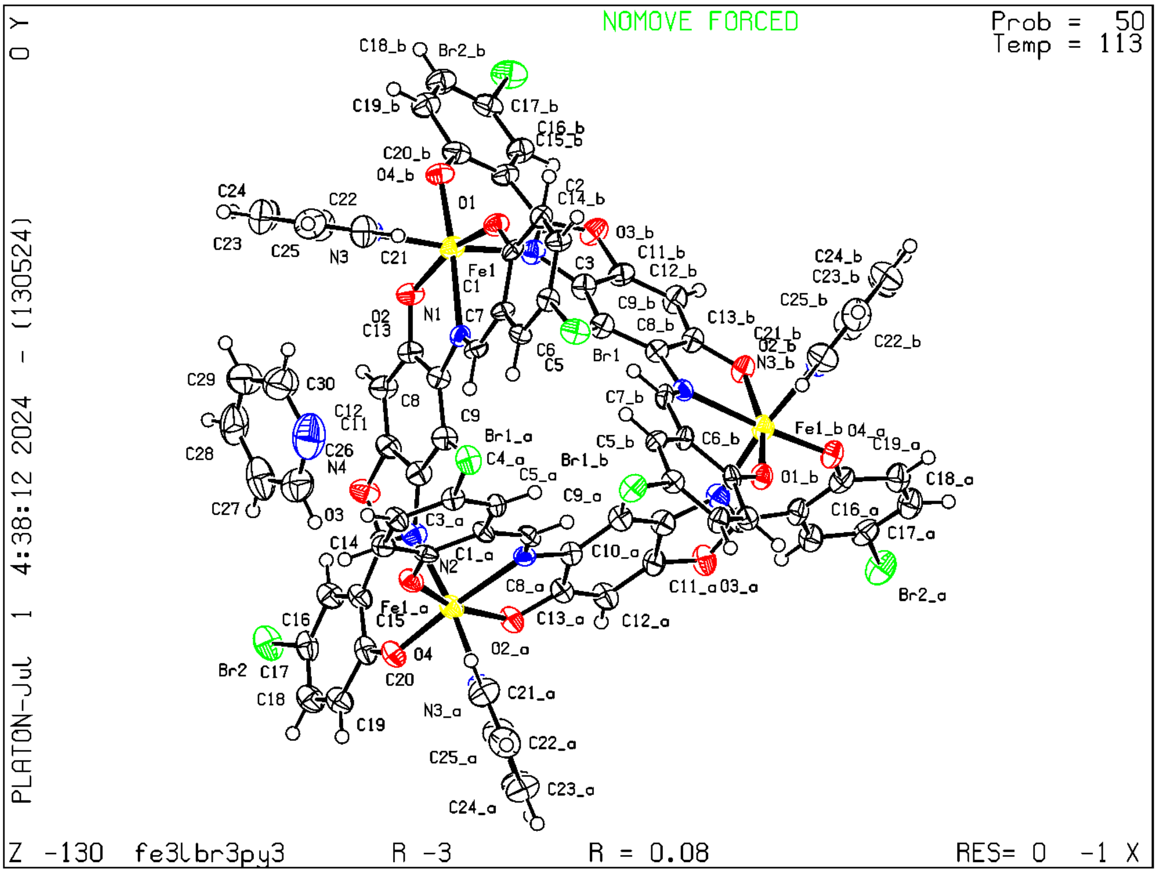

# checkCIF/PLATON report

Structure factors have been supplied for datablock(s) mn3l5br3py3

THIS REPORT IS FOR GUIDANCE ONLY. IF USED AS PART OF A REVIEW PROCEDURE FOR PUBLICATION, IT SHOULD NOT REPLACE THE EXPERTISE OF AN EXPERIENCED CRYSTALLOGRAPHIC REFEREE.

No syntax errors found. CIF dictionary Interpreting this report

# Datablock: mn3l5br3py3

---

Bond precision: C-C = 0.0078 A Wavelength=0.71075

| | | | |
|---|---|---|---|
| Cell: | a=15.995(3) | b=16.278(3) | c=17.829(4) |
| | alpha=78.264(9) | beta=83.109(9) | gamma=70.544(7) |
| Temperature: | 113 K | | |

| | Calculated | Reported |
|---|---|---|
| Volume | 4278.6(15) | 4278.8(15) |
| Space group | P -1 | P -1 |
| Hall group | -P 1 | -P 1 |
| Moiety formula | 2(C71 H41 Br6 Mn3 N8 O13), C5 H5 N [+ solvent] | C71 H41 Br6 Mn3 N8 O13, 0.5(C5 H5 N), 2.5[C5H5N] |
| Sum formula | C147 H87 Br12 Mn6 N17 O26 [+ solvent] | C86 H56 Br6 Mn3 N11 O13 |
| Mr | 3795.78 | 2095.69 |
| Dx,g cm-3 | 1.473 | 1.627 |
| Z | 1 | 2 |
| Mu (mm-1) | 3.296 | 3.306 |
| F000 | 1866.0 | 2076.0 |
| F000' | 1865.36 | |
| h,k,lmax | 20,21,23 | 20,21,23 |
| Nref | 19594 | 18867 |
| Tmin,Tmax | 0.515,0.673 | 0.550,0.729 |
| Tmin' | 0.494 | |

Correction method= # Reported T Limits: Tmin=0.550 Tmax=0.729
AbsCorr = NUMERICAL

Data completeness= 0.963 Theta(max)= 27.477

R(reflections)= 0.0602( 11076)    wR2(reflections)= 0.1645( 18867)

S = 0.984    Npar= 1014

---

The following ALERTS were generated. Each ALERT has the format
**test-name_ALERT_alert-type_alert-level**.
Click on the hyperlinks for more details of the test.

---

**Alert level B**

PLAT220_ALERT_2_B NonSolvent Resd 1 C Ueq(max)/Ueq(min) Range 6.6 Ratio

**Author Response: This alert refers to the carbon of the coordinated methanol. Around this methanol is small space, what allows larger thermal motion of the methanol carbon atoms. This leads to enlargement of displacement ellipsoids, in comparison to the other ligand.**

PLAT420_ALERT_2_B D-H Bond Without Acceptor O13 --H13 . Please Check

**Author Response: This alert refers to the hydroxyl group of the coordinated methanol. There is a small distance between this methanol and the adjacent molecule, and no hydrogen bonding sites are available.**

```
PLAT910_ALERT_3_B Missing # of FCF Reflection(s) Below Theta(Min).        27 Note
                  1  0  0,   2  0  0,  -1  1  0,   0  1  0,   1  1  0,   2  1  0,
                  0  2  0,   1  2  0,  -1 -1  1,   0 -1  1,   1 -1  1,  -1  0  1,
                  0  0  1,   1  0  1,   2  0  1,  -1  1  1,   0  1  1,   1  1  1,
                  2  1  1,   0  2  1,   1  2  1,   0 -1  2,  -1  0  2,   0  0  2,
```

**Author Response: Low-angle reflections rejected because of beam stop and high background scattering.**

---

**Alert level C**

```
ABSTY02_ALERT_1_C  An _exptl_absorpt_correction_type has been given without
            a literature citation. This should be contained in the
            _exptl_absorpt_process_details field.
            Absorption correction given as numerical
CRYSC01_ALERT_1_C The word below has not been recognised as a standard
            identifier.
            reddish
PLAT222_ALERT_3_C NonSolvent Resd 1  H   Uiso(max)/Uiso(min) Range        7.2 Ratio
PLAT230_ALERT_2_C Hirshfeld Test Diff for    C48      --C49      .        6.2 s.u.
PLAT234_ALERT_4_C Large Hirshfeld Difference O13      --C71      .       0.17 Ang.
PLAT242_ALERT_2_C Low    'MainMol' Ueq as Compared to Neighbors of        O13 Check
PLAT250_ALERT_2_C Large U3/U1 Ratio for <U(i,j)> Tensor(Resd    2)        3.3 Note
PLAT341_ALERT_3_C Low Bond Precision on  C-C Bonds ..............      0.0078 Ang.
PLAT431_ALERT_2_C Short Inter HL..A Contact  Br6      ..O3       .       3.21 Ang.
```

```
                                             1-x,-y,1-z  =      2_656 Check
PLAT906_ALERT_3_C Large K Value in the Analysis of Variance ......      6.130 Check
PLAT906_ALERT_3_C Large K Value in the Analysis of Variance ......      2.366 Check
PLAT911_ALERT_3_C Missing FCF Refl Between Thmin & STh/L=    0.600        255 Report
              -2  1  0,  -1  2  0,   4  3  0,   1  3  1,   2  2  2, -10 12  2,
              -9 13  2,  -8 14  2,   2 -3  3,   0 -2  3,  -3 -1  3,   2 -1  3,
               0  1  3, -13  9  3, -12 10  3, -11 11  3, -10 12  3,  -9 12  3,
              -9 13  3,  -8 13  3,  -8 14  3,  -2  0  4,  -1  1  4,   1  4  4,
             -12 10  4, -11 10  4, -11 11  4, -10 11  4, -10 12  4,  -9 12  4,
              -8 12  4,  -9 13  4,  -8 13  4,  -7 14  4,   2  4  5, -14  7  5,
             -13  8  5, -12  9  5, -11  9  5, -11 10  5, -10 10  5, -11 11  5,
             -10 11  5,  -9 11  5, -10 12  5,  -9 12  5,  -8 12  5,  -9 13  5,
              -8 13  5,  -7 14  5,   1 -5  6,   2 -4  6,  -2  2  6, -14  6  6,
             -13  7  6, -13  8  6, -12  8  6, -12  9  6, -11  9  6, -10  9  6,
             -11 10  6, -10 10  6,  -9 10  6, -10 11  6,  -9 11  6,  -9 12  6,
              -8 12  6,  -8 13  6,  -7 14  6,  15 -1  7,   1  1  7,   6  3  7,
             -14  5  7, -14  6  7, -13  6  7, -13  7  7, -12  7  7, -12  8  7,
             -11  8  7, -12  9  7, -11  9  7, -10  9  7,  -9  9  7, -11 10  7,
             -10 10  7,  -9 10  7, -10 11  7,  -9 11  7,  -9 12  7,  -8 12  7,
              -8 13  7,  -7 14  7, -14  5  8, -13  5  8, -13  6  8, -12  6  8,
PLAT971_ALERT_2_C Check Calcd Resid. Dens.  1.36Ang From N9            1.64 eA-3
```

---

## Alert level G

```
FORMU01_ALERT_2_G  There is a discrepancy between the atom counts in the
            _chemical_formula_sum and the formula from the _atom_site* data.
            Atom count from _chemical_formula_sum:C86 H56 Br6 Mn3 N11 O13
            Atom count from the _atom_site data:  C73.5 H43.5 Br6 Mn3 N8.5 O13
CELLZ01_ALERT_1_G Difference between formula and atom_site contents detected.
CELLZ01_ALERT_1_G ALERT: Large difference may be due to a
            symmetry error - see SYMMG tests
           From the CIF: _cell_formula_units_Z    2
           From the CIF: _chemical_formula_sum  C86 H56 Br6 Mn3 N11 O13
           TEST: Compare cell contents of formula and atom_site data

           atom    Z*formula  cif sites diff
           C        172.00    147.00   25.00
           H        112.00     87.00   25.00
           Br        12.00     12.00    0.00
           Mn         6.00      6.00    0.00
           N         22.00     17.00    5.00
           O         26.00     26.00    0.00
PLAT002_ALERT_2_G Number of Distance or Angle Restraints on AtSite         21 Note
PLAT003_ALERT_2_G Number of Uiso or U(i,j) Restrained non-H Atoms           6 Report
PLAT041_ALERT_1_G Calc. and Reported SumFormula    Strings  Differ     Please Check
              Calc: C73.50 H43.50 Br6 Mn3 N8.50 O13
              Rep.: C86 H56 Br6 Mn3 N11 O13
PLAT042_ALERT_1_G Calc. and Reported MoietyFormula Strings  Differ     Please Check
              Calc: 2(C71 H41 Br6 Mn3 N8 O13), C5 H5 N
              Rep.: C71 H41 Br6 Mn3 N8 O13, 0.5(C5 H5 N), 2.5[C5H5
                    N]
PLAT045_ALERT_1_G Calculated and Reported Z Differ by a Factor ...      0.500 Check
PLAT172_ALERT_4_G The CIF-Embedded .res File Contains DFIX Records          4 Report
PLAT173_ALERT_4_G The CIF-Embedded .res File Contains DANG Records          7 Report
PLAT174_ALERT_4_G The CIF-Embedded .res File Contains FLAT Records          3 Report
PLAT176_ALERT_4_G The CIF-Embedded .res File Contains SADI Records         15 Report
PLAT178_ALERT_4_G The CIF-Embedded .res File Contains SIMU Records          2 Report
```

```
PLAT187_ALERT_4_G The CIF-Embedded .res File Contains RIGU Records          2 Report
PLAT191_ALERT_3_G A Non-default SADI Restraint Value has been used     0.0400 Report
PLAT191_ALERT_3_G A Non-default SADI Restraint Value has been used     0.0400 Report
PLAT191_ALERT_3_G A Non-default SADI Restraint Value has been used     0.0400 Report
PLAT191_ALERT_3_G A Non-default SADI Restraint Value has been used     0.0400 Report
PLAT191_ALERT_3_G A Non-default SADI Restraint Value has been used     0.0400 Report
PLAT191_ALERT_3_G A Non-default SADI Restraint Value has been used     0.0400 Report
PLAT191_ALERT_3_G A Non-default SADI Restraint Value has been used     0.0400 Report
PLAT299_ALERT_4_G Atom Site Occupancy Constrained at ............         0.5 Check
              N9      C72     C73     C74     C75     C76     H72     H73
              H74     H75     H76
PLAT301_ALERT_3_G Main Residue  Disorder .............(Resd    1)          5% Note
PLAT302_ALERT_4_G Anion/Solvent/Minor-Residue Disorder (Resd    2)        100% Note
PLAT304_ALERT_4_G Non-Integer Number of Atoms in ..... (Resd    2)        5.50 Check
PLAT398_ALERT_2_G Deviating  C-O-C   Angle From 120 for O3       .       104.6 Degree
PLAT398_ALERT_2_G Deviating  C-O-C   Angle From 120 for O7       .       105.2 Degree
PLAT398_ALERT_2_G Deviating  C-O-C   Angle From 120 for O11      .       105.0 Degree
PLAT434_ALERT_2_G Short Inter HL..HL Contact Br2      ..Br3      .        3.55 Ang.
                                                x,y,-1+z  =      1_554 Check
PLAT606_ALERT_4_G Solvent Accessible VOID(S) in Structure ........          ! Info
PLAT789_ALERT_4_G Atoms with Negative _atom_site_disorder_group  #         11 Check
PLAT794_ALERT_5_G Tentative Bond Valency for Mn1       (I)       .        0.87 Info
PLAT794_ALERT_5_G Tentative Bond Valency for Mn3       (I)       .        0.89 Info
PLAT822_ALERT_4_G CIF-embedded .res Contains Negative PART Numbers          1 Check
PLAT860_ALERT_3_G Number of Least-Squares Restraints ............         126 Note
PLAT868_ALERT_4_G ALERTS Due to the Use of _smtbx_masks Suppressed          ! Info
PLAT912_ALERT_4_G Missing # of FCF Reflections Above STh/L=  0.600        446 Note
PLAT913_ALERT_3_G Missing # of Very Strong Reflections in FCF ....          1 Note
               -2  0  4,
PLAT933_ALERT_2_G Number of HKL-OMIT Records in Embedded .res File         10 Note
               -2  1  0,  -2  2  6,  -1  1  4,   0 -2  3,   0  1  3,   1  3  1,
                1  4  4,   2 -3  3,   2  2  2,   6  3  7,
PLAT941_ALERT_3_G Average HKL Measurement Multiplicity ..........         1.9 Low
PLAT969_ALERT_5_G The 'Henn et al.' R-Factor-gap value ..........       2.989 Note
              Predicted wR2: Based on SigI**2  5.50 or SHELX Weight 16.71
PLAT978_ALERT_2_G Number C-C Bonds with Positive Residual Density.          0 Info
```

---

```
   0 ALERT level A = Most likely a serious problem - resolve or explain
   3 ALERT level B = A potentially serious problem, consider carefully
  13 ALERT level C = Check. Ensure it is not caused by an omission or oversight
  42 ALERT level G = General information/check it is not something unexpected

   7 ALERT type 1 CIF construction/syntax error, inconsistent or missing data
  16 ALERT type 2 Indicator that the structure model may be wrong or deficient
  17 ALERT type 3 Indicator that the structure quality may be low
  15 ALERT type 4 Improvement, methodology, query or suggestion
   3 ALERT type 5 Informative message, check
```

---

---

It is advisable to attempt to resolve as many as possible of the alerts in all categories. Often the minor alerts point to easily fixed oversights, errors and omissions in your CIF or refinement strategy, so attention to these fine details can be worthwhile. In order to resolve some of the more serious problems it may be necessary to carry out additional measurements or structure refinements. However, the purpose of your study may justify the reported deviations and the more serious of these should normally be commented upon in the discussion or experimental section of a paper or in the "special_details" fields of the CIF. checkCIF was carefully designed to identify outliers and unusual parameters, but every test has its limitations and alerts that are not important in a particular case may appear. Conversely, the absence of alerts does not guarantee there are no aspects of the results needing attention. It is up to the individual to critically assess their own results and, if necessary, seek expert advice.

**Publication of your CIF in IUCr journals**

A basic structural check has been run on your CIF. These basic checks will be run on all CIFs submitted for publication in IUCr journals (*Acta Crystallographica*, *Journal of Applied Crystallography*, *Journal of Synchrotron Radiation*); however, if you intend to submit to *Acta Crystallographica Section C* or *E* or *IUCrData*, you should make sure that full publication checks are run on the final version of your CIF prior to submission.

**Publication of your CIF in other journals**

Please refer to the *Notes for Authors* of the relevant journal for any special instructions relating to CIF submission.

---

**PLATON version of 22/08/2024; check.def file version of 21/08/2024**

**Datablock mn3l5br3py3** - ellipsoid plot

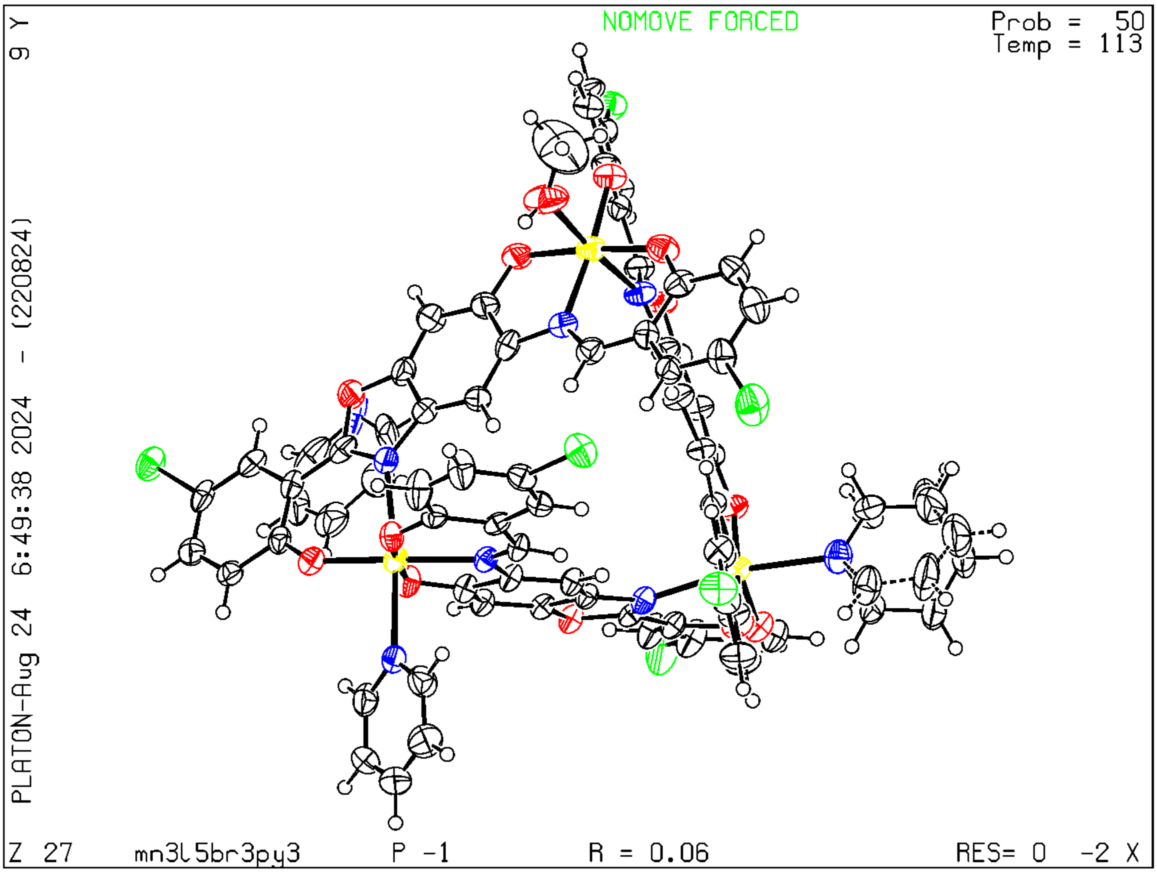

# checkCIF/PLATON report

Structure factors have been supplied for datablock(s) FeMn2L32Hdmf3

THIS REPORT IS FOR GUIDANCE ONLY. IF USED AS PART OF A REVIEW PROCEDURE FOR PUBLICATION, IT SHOULD NOT REPLACE THE EXPERTISE OF AN EXPERIENCED CRYSTALLOGRAPHIC REFEREE.

No syntax errors found.     CIF dictionary     Interpreting this report

# Datablock: FeMn2L32Hdmf3

---

```
Bond precision:   C-C = 0.0045 A          Wavelength=0.71075

Cell:           a=14.417(3)       b=15.001(3)       c=20.820(5)
                alpha=102.622(3)  beta=97.115(2)    gamma=94.088(3)
Temperature:    293 K
```

| | Calculated | Reported |
|---|---|---|
| Volume | 4337.5(16) | 4337.6(16) |
| Space group | P -1 | P -1 |
| Hall group | -P 1 | -P 1 |
| Moiety formula | C69 H54 Fe Mn2 N9 O15, C3 H7 N O [+ solvent] | C69 H54 Fe Mn2 N9 O15, C3 H7 N O, 1.5[C3H7NO] |
| Sum formula | C72 H61 Fe Mn2 N10 O16 [+ solvent] | C76.50 H71.50 Fe Mn2 N11.50 O17.50 |
| Mr | 1488.04 | 1597.68 |
| Dx,g cm-3 | 1.139 | 1.223 |
| Z | 2 | 2 |
| Mu (mm-1) | 0.512 | 0.519 |
| F000 | 1534.0 | 1654.0 |
| F000' | 1536.69 | |
| h,k,lmax | 18,19,27 | 18,19,26 |
| Nref | 19860 | 19007 |
| Tmin,Tmax | 0.786,0.838 | 0.812,0.860 |
| Tmin' | 0.784 | |

```
Correction method= # Reported T Limits: Tmin=0.812 Tmax=0.860
AbsCorr = MULTI-SCAN

Data completeness= 0.957            Theta(max)= 27.459
```

```
R(reflections)= 0.0624( 15754)                       wR2(reflections)=
                                                      0.1947( 19007)
S = 1.037                   Npar= 918
```

---

```
The following ALERTS were generated. Each ALERT has the format
       test-name_ALERT_alert-type_alert-level.
Click on the hyperlinks for more details of the test.
```

---

### Alert level B

```
PLAT241_ALERT_2_B High   'MainMol' Ueq as Compared to Neighbors of        O14 Check
```

**Author Response: The alerted atom is a coordinating atom bound to a transition metal. Its Ueq is larger compared to that of the metal element but is similar to the Ueq of other atoms within the ligand.**

```
PLAT910_ALERT_3_B Missing # of FCF Reflection(s) Below Theta(Min).         27 Note
                1  0  0,   2  0  0,  -1  1  0,   0  1  0,   1  1  0,  -1  2  0,
                0  2  0,   0 -2  1,   1 -2  1,  -1 -1  1,   0 -1  1,   1 -1  1,
               -2  0  1,  -1  0  1,   0  0  1,   1  0  1,  -1  1  1,   0  1  1,
                1  1  1,  -1 -1  2,   0 -1  2,   1 -1  2,  -1  0  2,   0  0  2,
```

**Author Response: Low-angle reflections rejected because of beam stop and high background scattering.**

---

### Alert level C

```
ABSTY02_ALERT_1_C  An _exptl_absorpt_correction_type has been given without
            a literature citation. This should be contained in the
            _exptl_absorpt_process_details field.
            Absorption correction given as multi-scan
PLAT029_ALERT_3_C _diffrn_measured_fraction_theta_full value Low .      0.975 Why?
PLAT220_ALERT_2_C NonSolvent   Resd 1  C   Ueq(max)/Ueq(min) Range        5.0 Ratio
PLAT220_ALERT_2_C NonSolvent   Resd 1  N   Ueq(max)/Ueq(min) Range        3.4 Ratio
PLAT222_ALERT_3_C NonSolvent Resd 1  H   Uiso(max)/Uiso(min) Range        5.9 Ratio
PLAT230_ALERT_2_C Hirshfeld Test Diff for    O14      --C66      .        6.7 s.u.
PLAT234_ALERT_4_C Large Hirshfeld Difference N8       --C65      .       0.20 Ang.
PLAT234_ALERT_4_C Large Hirshfeld Difference N8       --C66      .       0.18 Ang.
PLAT241_ALERT_2_C High   'MainMol' Ueq as Compared to Neighbors of         O5 Check
```

**Author Response: The alerted atom is a coordinating atom bound to a transition metal. Its Ueq is larger compared to that of the metal element but is similar to the Ueq of other atoms within the ligand.**

```
PLAT241_ALERT_2_C High   'MainMol' Ueq as Compared to Neighbors of         O6 Check
```

**Author Response: The alerted atom is a coordinating atom bound to a transition metal. Its Ueq is larger compared to that of the metal element but is similar to the Ueq of other atoms within the ligand.**

```
PLAT241_ALERT_2_C High   'MainMol' Ueq as Compared to Neighbors of       O15 Check
```

**Author Response: The alerted atom is a coordinating atom bound to a transition metal. Its Ueq is larger compared to that of the metal element but is similar to the Ueq of other atoms within the ligand.**

```
PLAT242_ALERT_2_C Low    'MainMol' Ueq as Compared to Neighbors of       Mn1 Check
PLAT242_ALERT_2_C Low    'MainMol' Ueq as Compared to Neighbors of        N7 Check
PLAT242_ALERT_2_C Low    'MainMol' Ueq as Compared to Neighbors of        N8 Check
PLAT242_ALERT_2_C Low    'MainMol' Ueq as Compared to Neighbors of        N9 Check
PLAT242_ALERT_2_C Low    'MainMol' Ueq as Compared to Neighbors of       C66 Check
PLAT244_ALERT_4_C Low    'Solvent' Ueq as Compared to Neighbors of       N10 Check
PLAT244_ALERT_4_C Low    'Solvent' Ueq as Compared to Neighbors of       C72 Check
PLAT260_ALERT_2_C Large Average Ueq of Residue Including      O16      0.141 Check
PLAT906_ALERT_3_C Large K Value in the Analysis of Variance ......     3.201 Check
PLAT911_ALERT_3_C Missing FCF Refl Between Thmin & STh/L=    0.600       362 Report
               -6  1  0,  -3  1  0,   3  1  0,  -6  3  0,  -2  3  0,   0  3  0,
               -2  4  0,   3  4  0,   4  4  0,  -3  5  0,  15  6  0, -16  7  0,
               -6  7  0,  14  7  0,  12  8  0,  13  8  0,  14  8  0,  11  9  0,
               12  9  0,  13  9  0,  11 10  0,  12 10  0,  13 10  0,  10 11  0,
               11 11  0,  12 11  0,  10 12  0,  11 12  0,   9 13  0,  10 13  0,
               -9-14  1, -10-13  1,  -6-13  1, -11-12  1, -10-12  1, -12-11  1,
              -11-11  1, -13-10  1, -12-10  1, -11-10  1, -13 -9  1, -12 -9  1,
              -11 -9  1, -14 -8  1, -13 -8  1, -12 -8  1,   3 -8  1, -15 -7  1,
              -14 -7  1, -13 -7  1,   3 -7  1, -15 -6  1,   4 -6  1, -16 -5  1,
                1 -4  1,   2 -4  1,   2 -3  1,   3 -3  1,  -3 -2  1,  -1 -2  1,
               -6 -1  1,  -3  0  1,   2  0  1,   3  0  1,  -2  1  1,   1  2  1,
               -4  4  1,   2  4  1,  15  6  1,  14  7  1,  12  8  1,  13  8  1,
               14  8  1,  11  9  1,  12  9  1,  13  9  1,  10 10  1,  11 10  1,
               12 10  1,  10 11  1,  11 11  1,   9 12  1,  10 12  1,  11 12  1,
                9 13  1, -10-13  2, -11-12  2, -12-11  2, -11-11  2, -13-10  2,
              -12-10  2, -11-10  2, -14 -9  2, -13 -9  2, -12 -9  2, -14 -8  2,
PLAT913_ALERT_3_C Missing # of Very Strong Reflections in FCF ....         8 Note
                0  1  0,  -1 -1  1,   1  0  1,  -1  1  2,   2 -3  3,  -3 -2  3,
               -1  2  4,  -1  0  5,
PLAT918_ALERT_3_C Reflection(s) with I(obs) much Smaller I(calc) .         2 Check
```

---

**Alert level G**

```
FORMU01_ALERT_2_G  There is a discrepancy between the atom counts in the
            _chemical_formula_sum and the formula from the _atom_site* data.
            Atom count from _chemical_formula_sum:C76.5 H71.5 Fe1 Mn2 N11.5 O17.5
            Atom count from the _atom_site data:  C72 H61 Fe1 Mn2 N10 O16
CELLZ01_ALERT_1_G Difference between formula and atom_site contents detected.
CELLZ01_ALERT_1_G ALERT: Large difference may be due to a
            symmetry error - see SYMMG tests
           From the CIF: _cell_formula_units_Z    2
           From the CIF: _chemical_formula_sum  C76.50 H71.50 Fe Mn2 N11.50 O17.50
           TEST: Compare cell contents of formula and atom_site data
```

```
          atom    Z*formula  cif sites diff
          C        153.00    144.00    9.00
          H        143.00    122.00   21.00
          Fe         2.00      2.00    0.00
          Mn         4.00      4.00    0.00
          N         23.00     20.00    3.00
          O         35.00     32.00    3.00
PLAT003_ALERT_2_G Number of Uiso or U(i,j) Restrained non-H Atoms           2 Report
PLAT041_ALERT_1_G Calc. and Reported SumFormula    Strings  Differ     Please Check
             Calc: C72 H61 Fe Mn2 N10 O16
             Rep.: C76.50 H71.50 Fe Mn2 N11.50 O17.50
PLAT042_ALERT_1_G Calc. and Reported MoietyFormula Strings  Differ     Please Check
             Calc: C69 H54 Fe Mn2 N9 O15, C3 H7 N O
             Rep.: C69 H54 Fe Mn2 N9 O15, C3 H7 N O, 1.5[C3H7NO]
PLAT072_ALERT_2_G SHELXL First  Parameter in WGHT  Unusually Large       0.11 Report
PLAT178_ALERT_4_G The CIF-Embedded .res File Contains SIMU Records          2 Report
PLAT187_ALERT_4_G The CIF-Embedded .res File Contains RIGU Records          2 Report
PLAT199_ALERT_1_G Reported _cell_measurement_temperature ..... (K)        293 Check
PLAT200_ALERT_1_G Reported   _diffrn_ambient_temperature ..... (K)        293 Check
PLAT232_ALERT_2_G Hirshfeld Test Diff (M-X)  Fe1      --O13      .        5.7 s.u.
PLAT232_ALERT_2_G Hirshfeld Test Diff (M-X)  Mn1      --O6       .        5.5 s.u.
PLAT232_ALERT_2_G Hirshfeld Test Diff (M-X)  Mn1      --O14      .       14.7 s.u.
PLAT232_ALERT_2_G Hirshfeld Test Diff (M-X)  Mn1      --N2       .        5.8 s.u.
PLAT232_ALERT_2_G Hirshfeld Test Diff (M-X)  Mn2      --O8       .        5.7 s.u.
PLAT232_ALERT_2_G Hirshfeld Test Diff (M-X)  Mn2      --O15      .       15.1 s.u.
PLAT398_ALERT_2_G Deviating  C-O-C   Angle From 120 for O3       .      104.2 Degree
PLAT398_ALERT_2_G Deviating  C-O-C   Angle From 120 for O7       .      104.7 Degree
PLAT398_ALERT_2_G Deviating  C-O-C   Angle From 120 for O11      .      104.9 Degree
PLAT606_ALERT_4_G Solvent Accessible VOID(S) in Structure .......           ! Info
PLAT794_ALERT_5_G Tentative Bond Valency for Fe1       (III)     .       3.13 Info
PLAT794_ALERT_5_G Tentative Bond Valency for Mn1       (I)       .       0.87 Info
PLAT794_ALERT_5_G Tentative Bond Valency for Mn2       (I)       .       0.85 Info
PLAT860_ALERT_3_G Number of Least-Squares Restraints ............          30 Note
PLAT868_ALERT_4_G ALERTS Due to the Use of _smtbx_masks Suppressed          ! Info
PLAT912_ALERT_4_G Missing # of FCF Reflections Above STh/L=  0.600        462 Note
PLAT933_ALERT_2_G Number of HKL-OMIT Records in Embedded .res File          5 Note
              -2 -1  3,  -2  0  3,  -1  1  3,   0  3  2,   1  2  1,
PLAT941_ALERT_3_G Average HKL Measurement Multiplicity ..........         1.9 Low
PLAT969_ALERT_5_G The 'Henn et al.' R-Factor-gap value ..........       6.825 Note
             Predicted wR2: Based on SigI**2  2.85 or SHELX Weight 18.77
PLAT978_ALERT_2_G Number C-C Bonds with Positive Residual Density.          0 Info
```

---

  0 **ALERT level A** = Most likely a serious problem - resolve or explain
  2 **ALERT level B** = A potentially serious problem, consider carefully
 23 **ALERT level C** = Check. Ensure it is not caused by an omission or oversight
 31 **ALERT level G** = General information/check it is not something unexpected

  7 ALERT type 1 CIF construction/syntax error, inconsistent or missing data
 27 ALERT type 2 Indicator that the structure model may be wrong or deficient
  9 ALERT type 3 Indicator that the structure quality may be low
  9 ALERT type 4 Improvement, methodology, query or suggestion
  4 ALERT type 5 Informative message, check

---

# checkCIF publication errors

---

**Alert level A**

```
PUBL004_ALERT_1_A  The contact author's name and address are missing,
            _publ_contact_author_name and _publ_contact_author_address.
PUBL005_ALERT_1_A  _publ_contact_author_email, _publ_contact_author_fax and
            _publ_contact_author_phone are all missing.
            At least one of these should be present.
PUBL006_ALERT_1_A  _publ_requested_journal is missing
            e.g. 'Acta Crystallographica Section C'
PUBL008_ALERT_1_A  _publ_section_title is missing. Title of paper.
PUBL009_ALERT_1_A  _publ_author_name is missing. List of author(s) name(s).
PUBL010_ALERT_1_A  _publ_author_address is missing. Author(s) address(es).
PUBL012_ALERT_1_A  _publ_section_abstract is missing.
            Abstract of paper in English.
```

---

```
   7 ALERT level A = Data missing that is essential or data in wrong format
   0 ALERT level G = General alerts. Data that may be required is missing
```

---

**Publication of your CIF**

You should attempt to resolve as many as possible of the alerts in all categories. Often the minor alerts point to easily fixed oversights, errors and omissions in your CIF or refinement strategy, so attention to these fine details can be worthwhile. In order to resolve some of the more serious problems it may be necessary to carry out additional measurements or structure refinements. However, the nature of your study may justify the reported deviations from journal submission requirements and the more serious of these should be commented upon in the discussion or experimental section of a paper or in the "special_details" fields of the CIF. *checkCIF* was carefully designed to identify outliers and unusual parameters, but every test has its limitations and alerts that are not important in a particular case may appear. Conversely, the absence of alerts does not guarantee there are no aspects of the results needing attention. It is up to the individual to critically assess their own results and, if necessary, seek expert advice.

If level A alerts remain, which you believe to be justified deviations, and you intend to submit this CIF for publication in a journal, you should additionally insert an explanation in your CIF using the Validation Reply Form (VRF) below. This will allow your explanation to be considered as part of the review process.

### Validation response form

Please find below a validation response form (VRF) that can be filled in and pasted into your CIF.

```
# start Validation Reply Form
_vrf_PUBL004_GLOBAL
;
PROBLEM: The contact author's name and address are missing,
RESPONSE: ...
```

```
;
_vrf_PUBL005_GLOBAL
;
PROBLEM: _publ_contact_author_email, _publ_contact_author_fax and
RESPONSE: ...
;
_vrf_PUBL006_GLOBAL
;
PROBLEM: _publ_requested_journal is missing
RESPONSE: ...
;
_vrf_PUBL008_GLOBAL
;
PROBLEM: _publ_section_title is missing. Title of paper.
RESPONSE: ...
;
_vrf_PUBL009_GLOBAL
;
PROBLEM: _publ_author_name is missing. List of author(s) name(s).
RESPONSE: ...
;
_vrf_PUBL010_GLOBAL
;
PROBLEM: _publ_author_address is missing. Author(s) address(es).
RESPONSE: ...
;
_vrf_PUBL012_GLOBAL
;
PROBLEM: _publ_section_abstract is missing.
RESPONSE: ...
;
# end Validation Reply Form
```

If you wish to submit your CIF for publication in Acta Crystallographica Section C or E, you should upload your CIF via the web. If you wish to submit your CIF for publication in IUCrData you should upload your CIF via the web. If your CIF is to form part of a submission to another IUCr journal, you will be asked, either during electronic submission or by the Co-editor handling your paper, to upload your CIF via our web site.

---

**PLATON version of 15/07/2024; check.def file version of 15/07/2024**

**Datablock FeMn2L32Hdmf3** - ellipsoid plot

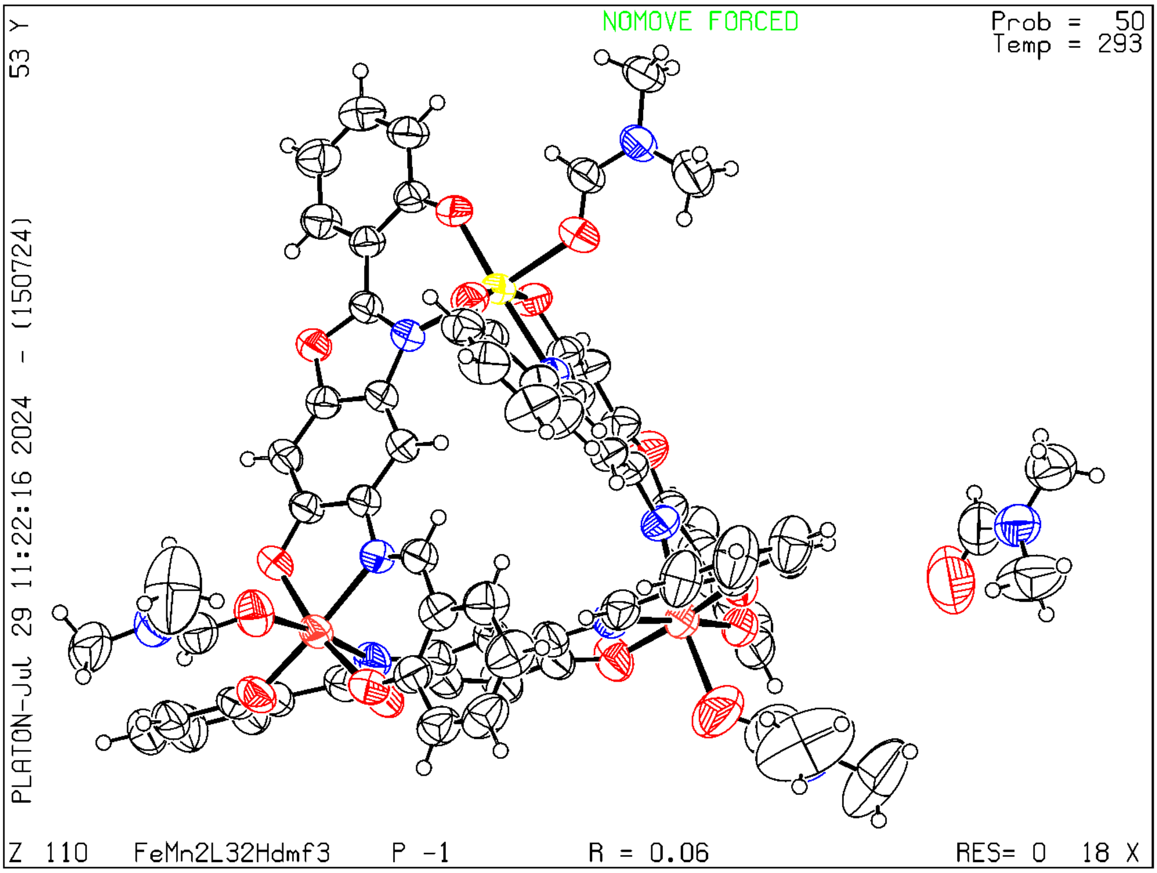

Supplement: Supplementary file 1 [file molecules-29-04307-s001.zip › Supplementary Materials.pdf]
